# Interval breast cancer characteristics before, during and after the transition from screen-film to full-field digital screening mammography

**DOI:** 10.1186/s12885-017-3294-5

**Published:** 2017-05-05

**Authors:** Rob M. G. van Bommel, Roy Weber, Adri C. Voogd, Joost Nederend, Marieke W. J. Louwman, Dick Venderink, Luc J. A. Strobbe, Matthieu J. C. Rutten, Menno L. Plaisier, Paul N. Lohle, Marianne J. H. Hooijen, Vivianne C.G. Tjan-Heijnen, Lucien E. M. Duijm

**Affiliations:** 1 Department of Radiology, Catharina Hospital, Michelangelolaan 2, 5623EJ Eindhoven, The Netherlands; 2 Department of Epidemiology, Maastricht University, P Debyelaan 1, 6229 HA Maastricht, The Netherlands; 3 Department of Research, Netherlands Comprehensive Cancer Organization (IKNL), PO Box 19079, 3501 DB Utrecht, The Netherlands; 4 Department of Radiology, Canisius Wilhelmina Hospital, Weg door Jonkerbos, 100 Nijmegen, The Netherlands; 5 Department of Surgery, Canisius-Wilhelmina Hospital, PO Box 9015, 6500 GS Nijmegen, The Netherlands; 6 Department of Radiology, Jeroen Bosch Hospital, Vlijmenseweg 10, 5223 GW ‘s-Hertogenbosch, The Netherlands; 7 Department of Radiology, Maxima Medical Centre, De Run 4600, 5504 DB Veldhoven, The Netherlands; 8 Department of Radiology, St Elisabeth Hospital, Hilvarenbeekseweg 60, 5022 GC Tilburg, The Netherlands; 9 Department of Radiology, St Anna Hospital, Bogardeind 2, 5664 EH Geldrop, The Netherlands; 10 Department of Internal Medicine, Division of Medical Oncology, GROW, Maastricht University Medical Centre, PO Box 5800, 6202 AZ Maastricht, The Netherlands; 11 Dutch Reference Centre for Screening, PO Box 6873, 6503GJ Nijmegen, The Netherlands

**Keywords:** Breast cancer, Mass screening, Mammography, Referral and consultation, Early detection of cancer

## Abstract

**Background:**

To determine the proportion of “true” interval cancers and tumor characteristics of interval breast cancers prior to, during and after the transition from screen-film mammography screening (SFM) to full-field digital mammography screening (FFDM).

**Methods:**

We included all women with interval cancers detected between January 2006 and January 2014. Breast imaging reports, biopsy results and breast surgery reports of all women recalled at screening mammography and of all women with interval breast cancers were collected. Two experienced screening radiologists reviewed the diagnostic mammograms, on which the interval cancers were diagnosed, as well as the prior screening mammograms and determined whether or not the interval cancer had been missed on the most recent screening mammogram. If not missed, the cancer was considered an occult (“true”) interval cancer.

**Results:**

A total of 442 interval cancers had been diagnosed, of which 144 at SFM with a prior SFM (SFM-SFM), 159 at FFDM with a prior SFM (FFDM-SFM) and 139 at FFDM with a prior FFDM (FFDM-FFDM). The transition from SFM to FFDM screening resulted in the diagnosis of more occult (“true”) interval cancers at FFDM-SFM than at SFM-SFM (65.4% (104/159) versus 49.3% (71/144), *P* < 0.01), but this increase was no longer statistically significant in women who had been screened digitally for the second time (57.6% (80/139) at FFDM-FFDM versus 49.3% (71/144) at SFM-SFM). Tumor characteristics were comparable for the three interval cancer cohorts, except of a lower porportion (75.7 and 78.0% versus 67.2% af FFDM-FFDM, *P* < 0.05) of invasive ductal cancers at FFDM with prior FFDM.

**Conclusions:**

An increase in the proportion of occult interval cancers is observed during the transition from SFM to FFDM screening mammography. However, this increase seems temporary and is no longer detectable after the second round of digital screening. Tumor characteristics and type of surgery are comparable for interval cancers detected prior to, during and after the transition from SFM to FFDM screening mammography, except of a lower proportion of invasive ductal cancers after the transition.

## Background

Full-field digital mammography (FFDM) has replaced screen-film mammography (SFM) in most regional and nation-wide breast cancer screening programs. Several studies have shown that the transition from screen-film screening to digital screening has increased cancer detection rates and program sensitivity, but it may also result in higher recall rates and more false-positive recalls [1–3]. Despite a higher cancer detection rate at FFDM, two screening studies reported a similar interval cancer rate at SFM and FFDM [4, 5]. Interval cancers are primary breast cancers that are diagnosed in women after a screening examination which has yielded a negative result, defined as no recommendation for recall or negative further assessment after recall, and before any subsequent screen is performed or within a time period equal to the screening interval (2 years) [6] Interval cancers are larger than screen detected cancers and have a worse prognosis [7–9].

Details on screening outcome at screen-film mammography screening and the 1st and 2nd round of digital screening have been published previously [3, 10]. The transition from screen-film to digital screening mammography may increase the proportion of ductal carcinoma in-situ (DCIS) and smaller invasive cancers at the first digital screening round [3, 4, 11], but does not appear to result in a change of the tumor characteristics of the interval cancers [4, 5]. However, the proportion of missed interval cancers among all interval cancers, and of interval cancers showing minimal signs at the most recent screening mammogram, were lower at digital than at screen-film screening. It is not known whether or not this effect remains present after the transition to FFDM (i.e., in women who undergo a second digital screening examination). In the current study we therefore determined interval cancer characteristics, tumor stage and surgical procedure prior to, during and after the transition from SFM to FFDM screening mammography.

## Methods

### Study population

The study was performed in a screening mammography region in the south of The Netherlands (BOZ, Bevolkings Onderzoek Zuid). The screening mammograms were obtained at four specialized units (one fixed unit and three mobile units). At these units, the transition from SFM to FFDM screening mammography took place in 2009-2010. We included all women with a subsequent screening examination and screened between January 2007 -January 2014. Women participating in our screening program are asked to give written permission for the use of their data for quality assurance and scientific purposes. Four women screened at our units did not give this permission and they were excluded from analysis. The Central Committee on Research Involving Human Subjects (CCMO) in The Hague, The Netherlands, waived ethical approval for this study.

### Screening procedure and recall

All Dutch women aged 50 to 75 years are offered biennial screening mammography. Further details of the Dutch Nation-wide Breast Cancer Screening Program have been described previously [12, 13]. In brief, all screen mammograms were obtained by a team of specialized screening mammography radiographers and all screens were double read by certified screening radiologists. Each screening radiologist evaluates at least 3000 screening mammograms yearly and all radiologists participate in clinical breast imaging.

During the evaluation of screening mammograms, the prior screening examination, whether SFM or FFDM, was always available for comparison. To facilitate this comparison, all screen-film mammograms that were used for comparison with digital screening mammography, were digitized using a film scanner and archiver designed for mammography (DigitalNow; R2/Hologic).

Each screen was given a BI-RADS score by the screening radiologists. Women with a BI-RADS 1 or 2 were not recalled and were invited for a subsequent biennial screen, whereas women with a BI-RAS score 0, 4 or 5 were recalled and evaluated at a breast unit at one of the 15 regional or university hospitals in the South of the Netherlands [14]. For each recall, the screening radiologist classified the abnormality visible at the screening mammogram in one of the following categories: suspicious mass, suspicious calcifications, suspicious mass with calcifications, architectural distortion, asymmetry or other abnormality.

### Diagnostic workup

After physical examination by the surgeon or dedicated breast nurse, additional mammographic views were obtained if necessary. All radiological findings were, again, classified according to the American College of Radiology BI-RADS [14]. Lesions classified as BI-RADS IV or V were routinely biopsied, whereas BI-RADS 3 lesions were either biopsied or followed-up at the discretion of the surgeon and/or radiologist. Dependent on the findings at physical examination and mammography, further diagnostic evaluation could include tomosynthesis, (3D) breast ultrasonography, magnetic resonance imaging, percutaneous fine needle aspiration cytology (FNAC), core needle biopsy (CNB), stereotactic biopsy or open surgical biopsy.

### Detection and review of interval cancers

During a follow-up period of 2 years (until the next biennial screen), all data on diagnostic imaging, clinical data, biopsy results, breast surgery reports and pathology reports of recalled women were collected. All data were stored into a computerized spreadsheet (Excel; Microsoft, Redmond, WA, USA). Interval cancers were defined as breast cancers diagnosed in women after a screening examination yielded negative results (no recommendation for recall) and before the subsequent biennial screen was performed. As a validated connection between the Dutch National comprehensive Cancer Centre and the Screening Information System was not available yet for our inclusion period, we traced interval cancers through linkage of all radiotherapy reports that were received from the regional radiotherapy institutes concerning women who underwent radiotherapy for breast malignancy with women who had participated in the screening program. Furthermore, we inquired about pathology specimens at the various regional pathology laboratories, some months after any hospital had requested the screening mammograms of a woman who had not been referred for further analysis. Also, the pathology records were obtained if a woman cancelled a call for subsequent screening because breast cancer had been diagnosed after a previous negative screen. Finally, we received the occasional reports on interval cancers provided by general practitioners or medical specialists to the screening centre.

All screening mammograms prior to the detection of an interval cancer, as well as the diagnostic mammogram obtained at the time of interval cancer detection, were reviewed by two experienced screening radiologists. Breast density was categorized according to the American College of Radiology (2003). The interval cancers were classified as missed, minimal sign [15] or occult on the most recent screening mammogram, according to the European guidelines [16]. Finally, when an abnormality was retrospectively visible at the latest screen at the site where the interval cancer had developed, this abnormality was categorized as a mass, microcalcifications, mass with calcifications, asymmetry, architectural distortion, or other mammographic abnormality. The two radiologists were blinded for each other’s review and consensus was reached for discrepant readings.

### Statistical analysis

The interval cancers (ICs) were divided into three cohorts, dependent on the technique used for the previous screening mammograms: 1) ICs diagnosed following two previous SFM screening rounds (SFM-SFM cohort); 2) ICs diagnosed following a most recent, subsequent FFDM screen, which was preceded by a SFM screen (FFDM-SFM cohort) and 3) ICs diagnosed after two subsequent FFDM screening rounds (FFDM-FFDM cohort). To compare differences in cancer characteristics between these three groups we used Z test, chi-square test Fisher’s exact test, whichever was appropriate. An independent samples *T*-test wasperformed for the comparison of means (e.g., tumor size). All tests were two-sided and the significance level was set at 5%. Statistical analyses were performed using Statistical Package for Social Sciences 22 (SPSS Inc. Chicago, IL) or SAS 9.4 (SAS Institute Inc., Cary, NC).

## Results

### Overall screening results

A total of 326,783 subsequent screens were obtained between January 1, 2007and January 1, 2014, with 2024 screen detected cancers and 442 interval cancers (Table 1). The overall recall rate was 2.2% (7305/326783), resulting in a cancer detection rate of 6.2 per 1000 screens (2024/326783) and a positive predictive value of 27.7% (2024/7305). Of the interval cancers, 144 were diagnosed in the SFM-SFM cohort, and respectively 159 and 139 in the FFDM-SFM and FFDM-FFDM cohorts. The three groups showed comparable proportions of invasive interval cancers versus interval DCIS (94.4% (136/144) at SFM-SFM, 94.3% (150/159) at FFDM-SFM and 94.2% (131/139) at FFDM-FFDM) (Table 1).

**Table 1:** Baseline characteristics of interval cancers at subsequent, biennial screening mammography

	A SFM with prior SFM	B FFDM with prior SFM	C FFDM with prior FFDM
Interval cancers, *n*	144	159	139
Ductal carcinoma in situ, *n* (%)	8 (5.6)	9 (5.7)	8 (5.8)
Invasive cancer, *n* (%)	136 (94.4)	150 (94.3)	131 (94.2)
Visibility of interval cancer at latest screen			
Missed, *n* (%)	42 (29.2)	35 (22.0)	39 (28.1)
Minimal sign, *n* (%)	31* (21.5)	20 (12.6)	20 (14.4)
Occult, *n* (%)	71^†^ (49.3)	104 (65.4)	80 (57.6)
Interval between latest screen and interval cancer			
≤ 1 year, n (%)	52 (36.1)	59 (37.1)	53 (38.1)
> 1 year, n (%)	92 (63.9)	100 (62.9)	86 (61.8)

*SFM* screen-film mammography, *FFDM* full-field digital mammography

* significantly different from B (*p* < 0.05)

^†^ significantly different from B (*p* < 0.01)

### Prior visibility and mammographic characteristics of interval cancers

A significantly larger proportion of interval cancers at subsequent screening mammography was considered occult (so-called true interval cancers) at the first digital screening round than at screen-film screening (65.4% at FFDM-SFM versus 49.3% at SFM-SFM, *P* < 0.01, Table 1). This proportion, however, decreased to 57.6% at the 2nd digital screening round, which was no longer statistically significantly different when compared to the proportion observed at screen-film screening. The proportions of interval cancers, detected either in the first year or second year following a negative screen (i.e., no recommendation for recall), were similar for the three screening cohorts, with respectively 63.9% (SFM-SFM), 62.9% (FFDM-SFM) and 61.8% (FFDM-FFDM) of interval cancers diagnosed more than one year after a negative screen (Table 1). The distribution of mammographic abnormalities, in the case of missed interval cancers and minimal sign interval cancers at the latest screening mammogram, was comparable for the three cohorts. A mass was present in 65.7%-69.2% and 50.0%-61.3% of missed interval cancers and minimal sign interval cancers, respectively (Table 2). Also, when looking separately at missed interval cancers, minimal sign interval cancers and occult interval cancers, we observed no significant differences in tumor size distribution, average tumor size or breast density at the latest screening mammogram between the three screening cohorts.

**Table 2:** Mammographic features of interval breast cancers at the latest screen and tumor size distribution

	A SFM with prior SFM	B FFDM with prior SFM	C FFDM with prior FFDM
Missed interval cancers at latest screen, *n*	42	35	39
Mammographic abnormality at latest screen, *n* (%)			
Mass	28 (66.7)	23 (65.7)	27 (69.2)
Calcifications	2 (4.8)	5 (14.3)	5 (12.8)
Mass with calcifications	5 (11.9)	4 (11.4)	4 (10.3)
Asymmetry	2 (4.8)	0 (0)	0 (0.0)
Architectural distortion	5 (11.9)	3 (8.6)	3 (7.7)
Type of interval cancer, *n* (%)			
Ductal carcinoma in-situ	1 (2.4)	1 (2.9)	2 (5.1)
Invasive cancer	41 (97.6)	34 (97.1)	37 (94.9)
Size distribution of invasive interval cancers, *n* (%)			
T1a-b	2 (4.9)	5 (14.7)	4 (10.8)
T1c	15 (36.6)	8 (23.5)	9 (24.3)
T2+	24 (58.5)	20 (58.8)	24 (64.9)
Unknown	0 (0.0)	1 (2.9)	0 (0.0)
Mean size of invasive interval cancers, mm (range)	29.3 (8-80)	28.8 (2-100)	25.5 (6-120)
Breast density at latest screen, *n* (%)			
ACR I & II	23 (54.8)	21 (60.0)	23 (59.0)
ACR III & IV	19 (45.2)	14 (40.0)	16 (41.0)
Minimal sign interval cancers at latest screen, *n*	31	20	20
Mammographic abnormality at latest screen, *n* (%)			
Mass	19 (61.3)	10 (50.0)	10 (50.0)
Calcifications	7 (22.6)	2 (10.0)	2 (10.0)
Mass with calcifications	0 (0.0)	2 (10.0)	0 (0.0)
Asymmetry	3 (9.7)	1 (5.0)	5 (25.0)
Architectural distortion, *n* (%)	2 (6.5)	5 (25.0)	3 (15.0)
Type of interval cancer, *n* (%)			
Ductal carcinoma in-situ	2 (6.5)	2 (10.0)	1 (5.0)
Invasive cancer	29 (93.5)	18 (90.0)	19 (95.0)
Size distribution of invasive interval cancers, *n* (%)			
T1a-b	2 (6.9)	0 (0.0)	1 (5.3)
T1c	13 (44.8)	8 (44.4)	7 (36.8)
T2+	14 (48.3)	10 (55.6)	11 (57.9)
Mean size of invasive interval cancers, mm (range)	24.5 (1-60)	29.1 (11-80)	28.4 (5-60)
Breast density at latest screen, *n* (%)			
ACR I & II	21 (67.7)	14 (70.0)	11 (55.0)
ACR III & IV	10 (32.2)	6 (30.0)	9 (45.0)
Occult interval cancers at latest screen	71	104	80
Type of interval cancer, *n* (%)			
Ductal carcinoma in-situ	5 (7.0)	6 (5.8)	5 (6.3)
Invasive cancer	66 (93.0)	98 (94.2)	75 (93.8)
Size distribution of invasive interval cancers, *n* (%)			
T1a-b	12 (18.2)	15 (15.3)	12 (16.0)
T1c	22 (33.3)	34 (34.7)	26 (34.7)
T2+	32 (48.5)	48 (49.0)	33 (44.0)
Unknown	0 (0.0)	1 (1.0)	4 (5.3)
Mean size of invasive interval cancers, mm (range)	21.8 (2-60)	25.3 (2-90)	23.2 (3-95)
Breast density at latest screen, *n* (%)			
ACR I & II	38 (53.5)	65 (62.5)	44 (55.0)
ACR III & IV	33 (46.5)	39 (37.5)	36 (45.0)

*SFM* screen-film mammography, *FFDM* full-field digital mammography, *ACR* American College of Radiology

### Tumor stage and tumor biology characteristics of interval cancers

The distribution of the histological grade of interval ductal carcinoma in-situ was comparable for the three screening cohorts (Table 3). Tumor characteristics of interval cancers were comparable for the 3 subgroups, except of a larger porportion of invasive ductal cancers at subsequent SFM screening mammography . For invasive interval cancers, we neither found significant differences in tumor stage, Bloom and Richardson (B&R) distribution or receptor status. Invasive ductal cancer was by far the most common histological subtype (respectively 75.7% at SFM-SFM, 78.0% at FFDM-SFM and 67.2% at FFDM-FFDM), about half of the invasive cancers were >20 mm (T2+) in each cohort and the proportion of lymph-node positive cancers ranged from 41.2% to 45.6%. A majority of invasive interval cancers were graded B&R I/II (respectively 74.3% at SFM-SFM (101/136), 76.7% at FFDM-SFM (115/150) and 77.1% at FFDM-FFDM (101/131)).

**Table 3:** Tumor characteristics of interval breast cancers

	A SFM with prior SFM	B FFDM with prior SFM	C FFDM with prior FFDM
Ductal carcinoma in-situ			
Grade, *n* (%)			
Low	2 (25.0)	3 (33.3)	1 (12.5)
Intermediate	2 (25.0)	3 (33.3)	3 (37.5)
High	4 (50.0)	3 (33.3)	4 (50.0)
Invasive cancer			
Type, *n* (%)			
Ductal	103 (75.7)*	117 (78.0)^†^	88 (67.2)
Lobular	23 (16.9)	19 (12.7)	28 (21.4)
Mixed ductal/lobular	7 (5.1)	7 (4.7)	7 (5.3)
Other	3 (2.2)	7 (4.7)	8 (6.1)
Stage, *n* (%)			
T1a-c	66 (48.5)	70 (46.7)	59 (45.0)
T2+	70 (51.5)	78 (52.0)	68 (51.9)
Unknown	0 (0.0)	2 (1.3)	4 (3.1)
Lymph node status, n (%)			
N+	62 (45.6)	63 (42.0)	54 (41.2)
No	73 (53.7)	84 (56.0)	73 (55.7)
Unknown	1 (0.7)	3 (2.0)	4 (3.1)
Bloom & Richardson grade, *n* (%)			
I	44 (32.4)	38 (25.3)	33 (25.2)
II	57 (41.9)	77 (51.3)	68 (51.9)
III	31 (22.8)	30 (20.0)	29 (22.1)
Unknown	4 (2.9)	5 (3.3)	1 (0.8)
Estrogen receptor, *n* (%)			
Positive	101 (74.3)	123 (82.0)	106 (80.9)
Negative	35 (25.7)	27 (18.0)	25 (19.1)
Progesteron receptor, *n* (%)			
Positive	74 (54.4)	87 (58.0)	76 (58.0)
Negative	62 (45.6)	63 (42.0)	55 (42.0)
Her2/Neu receptor, *n* (%)			
Positive	19 (14.0)	22 (14.7)	14 (10.7)
Negative	116 (85.3)	127 (84.7)	117 (89.3)
Unknown	1 (0.7)	1 (0.7)	0 (0.0)
Triple receptor-negative, *n* (%)	25 (18.5)	21 (14.0)	17 (13.0)

* significantly different from C (*p* < 0.05)

^†^ significantly different from C (*p* < 0.01)

### Surgical treatment of interval cancers

We observed no significant differences in the surgical treatment (either breast conserving surgery or mastectomy) of interval cancers in the three groups, with a majority of women being treated with breast conserving surgery (68.8% (99/144) at SFM-SFM, 63.5% (101/159) at FFDM-SFM and 65.5% (91/139) at FFDM-FFDM).

## Discussion

In the current study we determined the characteristics of interval cancers before, during and after transition from screen-film mammography to full-field digital mammography. During the transition at the first digital screening round, we observed a decreased proportion of missed interval cancers and interval cancers showing a minimal sign at the latest screen. This decrease was slightly lower in the second round of digital screening (57.6% versus 65.4%), but no longer statistically significantly different. Tumor stage, tumor biology and surgical treatment were comparable for the three interval cancer cohorts.

Data on interval cancers in the era of digital screening mammography are sparse, especially those related to interval cancers diagnosed after repetitive rounds of digital screening mammography. We previously reported a higher cancer detection rate during the transition from screen-film to full-field digital screening mammography [5], and this finding persisted in the second round of digital screening [10]. This increased detection rate, however, came along with more false-positive screen results and therefore a decreased positive predictive value of recall. In the current study, we found that a significantly higher proportion of interval cancers at the first round of digital screening were occult on the latest subsequent screening mammogram than at screen-film screening. A smaller Norwegian study, however, reported similar proportions of occult interval cancers at screen-film screening and the first round of digital screening [4]. This contradictory finding may be partly explained by differences in study population (the Norwegian study included women aged 50-69 years and was not limited to subsequent screens), reading strategy and screening outcome parameters (including lower recall rates and a higher positive predictive value of recall in our study). In our study, the decrease in proportion of occult interval cancers was slightly lower after the second round of digital screening and no longer statistically significant. We do not have a straightforward explanation for this observation as we expected the presence of a learning curve during the transition which would result in a persistent larger proportion of true interval cancers at repeated digital screening. This insinuates a steeper learning curve than expected beforehand. The overall superior technique of digital mammography at the first digital screening round may have resulted in the detection of breast cancers that otherwise would have resulted in interval cancers considered to be missed at review.

A previous study found some more asymmetries and less calcifications in missed interval cancers diagnosed after the first digital screening round than after screen-film screening, but these differences were not statistically significant [4]. Although digital mammography may improve the detection of grouped calcifications and densities with calcifications [1, 17–21], we also observed a similar distribution of mammographic abnormalities for missed interval cancers and interval cancers showing minimal signs at the latest screening examination.

The proportion of DCIS among all interval cancers was comparable for the three screened cohorts, which is in line with previous findings [11]. Another study reported a significantly smaller tumor size at digital than at screen-film screening mammography for invasive interval cancers presenting as a mass [4]. However, we did not observe any significant differences in mean tumor size, local tumor stage or lymph node stage among the different screening groups. This difference can probably be explained by the facts that we measured tumor size on the surgical specimen and not on imaging and the lack of sample size.

To our knowledge, no previous studies have compared tumor biology characteristics (e.g., histological subtype and receptor status) and surgical treatment of interval cancers diagnosed before, during and after introduction of digital screening mammography. Again, we found no significant differences between the screened cohorts with respect to these outcome parameters. We observed a relatively high proportion of lobular carcinoma in all interval cancer cohorts, ranging from 12.7%-21.4%. Detection of lobular carcinoma remains a point of concern in the era of digital screening [22].

Our study has certain limitations. The design of the Dutch breast screening program may be different from those in other countries, which may limit extrapolation of our findings to other screening programs. In The Netherlands, women are offered biennial screening between 50 and 75 years of age, in accordance with many other European countries. However, screening programs in the US often offer annual screening before the age of 50, whereas the UK offers triennial screening. The Dutch program is characterized by the lowest recall rate among European screening programs, and all screening mammograms are routinely double read by two certified screening radiologists. Finally, the limited follow-up period, especially for interval cancers detected after the second digital screening round, did not allow us to draw conclusions on prognosis of survival between the three cohorts.

## Conclusions

In summary, we found that the higher proportion of true interval cancers during the introduction of digital screening declines after the second digital round and is no longer statistically significant. Tumor stage and tumor biology characteristics were comparable for interval cancers, whether detected after screen-film screening or detected after the first or second round of digital screening. This study shows that digital screening will probably not lessen the detrimental effect of interval cancers in screening mammography programs.

## Abbreviations

B&R: Bloom and Richardson classification
BI-RADS: Breast imaging reporting and data system
CCMO: The Central Committee on Research Involving Human Subjects
CNB: Core needle biopsy
DCIS: Ductal carcinoma in situ
FFDM: Full-field digital mammography
FNAC: Fine needle aspiration cytology
IC: Interval cancer
SFM: Screen-film mammography
UK: United Kingdom
US: United States of America
